# The Cortical Chlorenchyma Collaboration Gradient Dominates the Shoot Economics Space in *Larix principis-rupprechtii*

**DOI:** 10.3390/life15081310

**Published:** 2025-08-19

**Authors:** Yang Yu, Huayong Zhang, Zhongyu Wang, Zhao Liu

**Affiliations:** 1 Research Center for Engineering Ecology and Nonlinear Science, North China Electric Power University, Beijing 102206, China; 120192132243@ncepu.edu.cn (Y.Y.); zhy_wang@ncepu.edu.cn (Z.W.); 2 Theoretical Ecology and Engineering Ecology Research Group, School of Life Sciences, Shandong University, Qingdao 250100, China; liuzhao9555@sdu.edu.cn

**Keywords:** shoots, economic spectrum, collaboration gradient, cortical chlorenchyma, environmental responses

## Abstract

Plant economics is based on carbon and nutrients rather than money. While leaf strategies aboveground are well characterized along an economic spectrum from “fast-growing and short-lived” to “slow and conservative,” economic models defined by aboveground shoot strategies remain unclear. Here, we offer a comprehensive view of aboveground economics and show that collaboration between shoots and stem cortical chlorenchyma can break out of the one-dimensional economic spectrum, offering a full range of economic possibilities. Trait data from 1551 current-year shoots of a single species confirm the classical fast–slow “conservation” gradient but reveal that most variation is explained by an orthogonal “cooperation” gradient, ranging from self-reliant resource acquisition to outsourced nutrient synthesis via the stem cortical chlorenchyma. This expanded “shoot economics space” provides a solid foundation for predicting aboveground responses to environmental change.

## 1. Introduction

Plant functional traits are closely related to plant survival, growth, and reproduction, and can reflect their adaptive strategies [1]. Many previous studies on plant functional traits have typically used species as the unit of analysis [2,3,4,5,6,7,8]; however, under different environmental conditions, the same species may exhibit substantial intraspecific trait variation. Intraspecific trait variation can more sensitively reflect plant responses to abiotic environmental filtering and biotic interactions [9,10,11,12]. Therefore, assessing the patterns of intraspecific variation and covariation networks of plant functional traits can provide new insights to guide our understanding of plant survival and adaptation strategies.

In the search for general patterns, ecologists have applied economic theory to explain variation in plant functional traits [5]. Among these, leaf trait variation was one of the earliest to be explored, as leaves are the organs through which aboveground plants acquire resources via photosynthesis [5,6,13]. Consequently, aboveground plant strategies are distributed along the “leaf economics spectrum” ref. [6], ranging from cheaply constructed but short-lived leaves (optimized for “fast” resource acquisition) to more expensive but long-lasting leaves (offering “slower” returns over longer timescales).

As the most active fundamental units of the aboveground branching system in woody plants [14,15,16], current-year shoots are physically closely connected to leaves and acquire resources from them and are often regarded as the aboveground equivalent of leaves [17,18]. Therefore, variation in fine-branch traits has been thought to follow a similar one-dimensional spectrum [5,19]. At one end of this spectrum, plants with “fast” aboveground resource acquisition strategies are expected to construct long, narrow-diameter branches that require minimal biomass investment but exhibit high metabolic rates [20,21]. At the other end, plants with “slow” strategies are expected to build thicker, denser branches to achieve longer lifespans and extended investment return periods [22,23].

However, mixed empirical results have led ecologists to question whether variation in branch traits can be fully explained by a one-dimensional “fast–slow” economic spectrum [24,25,26,27]. Instead, when considered collectively, early findings suggest that variation in branch traits may be driven by multiple evolutionary pressures [22,28,29,30]. Here, we aim to address this issue by proposing a new conceptual framework for branch economics that better captures the complexity of aboveground resource acquisition strategies. First, we integrated existing knowledge to construct a conceptual understanding of the covariation among five key shoot traits. Second, we tested our conceptual model using functional trait data from 1551 shoots of a single tree species. Third, we investigated the generality of this concept across different branch order levels. All analyses were conducted using newly collected fine-branch trait data.

## 2. Materials and Methods

### 2.1. Study Species

As a dominant species in cold temperate coniferous forests, *Larix principis-rupprechtii* is mainly distributed in mid- to high-altitude regions of northern and central China [31]. *Larix principis-rupprechtii* is a strongly light-demanding species that thrives in upper canopies or forest gaps due to its high dependence on sunlight. Seedlings can grow normally under intense light, while shading significantly suppresses their growth and reduces chlorophyll content. This species exhibits strong apical dominance and possesses a highly competitive ability for light resources [32]. *Larix principis-rupprechtii* is relatively sensitive to water availability and grows best in regions with annual precipitation between 600 and 1000 mm. It has strong cold tolerance and can withstand extremely low temperatures below −30 °C, with an optimal growth temperature of 10–20 °C. It prefers deep, fertile, moist, and well-drained acidic loam or sandy loam soils, with a suitable pH range of 4.5–6.5. It is intolerant of saline–alkali and heavy clay soils and is sensitive to phosphorus and nitrogen levels in the soil—nutrient deficiencies can limit the growth of its branches and leaves. Due to its high timber value and cold resistance, it is commonly used in afforestation projects in northern China [33].

### 2.2. Study Site

**Figure 1:**
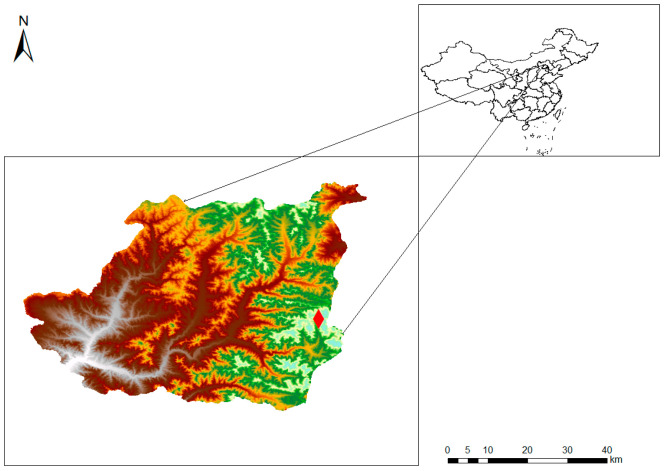
Study area and sampling location (red diamond marker indicates sampling point).

The study area is located approximately 24 km northeast of *Chongli* District, *Zhangjiakou* City, Hebei Province, within the core zone of the 2022 *Winter Olympics* ecological restoration and water conservation function enhancement demonstration area. The specific location ranges from 40°52′12″ to 40°58′48″ N and from 115°19′48″ to 115°31′12″ E. The total area of the demonstration zone is 118 km^2^, as shown in Figure 1. This comprehensive demonstration area falls within the East Asian continental monsoon climate zone. Winters are cold with low precipitation, frequent cold air activity, and strong winds. Summers are warm with rapidly rising temperatures and prone to heavy rainstorms. The average winter temperature is −12 °C, while the average summer temperature is 18.4 °C. The annual mean temperature is 7.5 °C, with an extreme high of 42 °C and an extreme low of −34.7 °C. The maximum wind speed can reach 20 m/s. The area experiences early snowfall, thick snow cover, and a long snow retention period, with cumulative winter snow depth reaching around 1 m. The average frost-free period exceeds 150 days. The region has an average annual precipitation of about 426 mm, with highly uneven temporal distribution. Around 80% of the annual rainfall occurs between June and September, mostly in the form of localized heavy storms and hail. There is also substantial interannual variation in precipitation, with alternating years of abundance and drought [34].

### 2.3. Sampling Procedures

A 20 × 20 m plot was established within a *Larix principis-rupprechtii* plantation. In early August 2019, the height and diameter at breast height (DBH) of every tree within the plot were recorded. Three larch saplings, each 5.5–6.0 m tall, were randomly selected. At the beginning of the study, branch systems from the upper crown of each tree were chosen to minimize variation in branch size. A total of 29 branch systems were selected—10, 10, and 9 from each tree, respectively. Two-dimensional diagrams were drawn to describe the branching structure of each branch system. Because *Larix principis-rupprechtii* has horizontally orientated shoots with planar leaf arrangements on vertical stems, all branches within a first-order branch system lie nearly in a horizontal plane. To analyze trait variation and coordination patterns associated with branch order, the order number of each terminal branch was determined from the structural diagrams based on centrifugal ranking (rather than centripetal ranking) [35,36].

**Figure 2:**
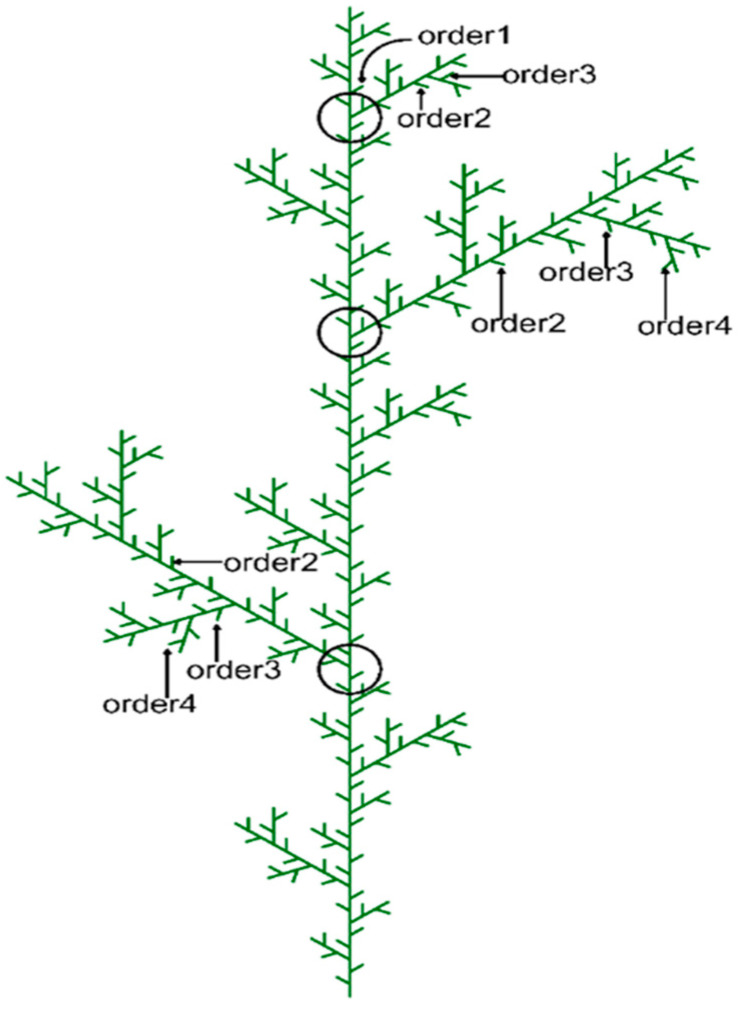
Diagram of the branching structure of *Larix principis-rupprechtii* and branch ordering in this study.

The branch order of each axis was determined. The main stem was defined as the zero-order axis; branches emerging directly from the main stem were classified as first-order axes; branches growing on the first-order axes were defined as second-order axes; and so on (Figure 2). In this study, the term “axis” is used to refer to each linear branch continuum of the same order. The term “branch system” is reserved for the set of lateral axes borne on a primary axis; that is, a branch system represents the collection of axes composed of the first-order axis and all its lateral second-, third-, and fourth-order axes [35,37,38].

### 2.4. Trait Measurements

We measured functional traits from 29 complete branch systems. Specifically, we focused on the length (SL) and diameter (SD) of current-year shoots produced on terminal branches. The diameter of current-year shoots across all branch orders was measured using a caliper. For shoot length, relatively short segments were measured with a caliper, while longer segments were measured with a measuring tape. All current-year shoot segments were oven-dried at 65 °C for 48 h and then weighed (±0.01 g). The cross-section of current-year shoots was assumed to be cylindrical. Stem tissue density (STD) was calculated as the ratio of shoot dry mass to its volume [27], and specific stem length (SSL) was calculated as the shoot length divided by dry mass [20]. After drying, the shoots were ground into fine powder, and nitrogen concentration (N, mg/g) was measured using an elemental analyzer (Vario EL III, Elementar, Hanau, Germany).

### 2.5. Data Analysis

We first assessed the bivariate relationships among five core functional traits (SD, SL, SSL, STD, and N) to build our conceptual framework. We used principal component analysis (PCA) to identify the main axes of variation in branch economic traits. PCA was conducted on the five core traits using the “*factoextra*” function from the “*FactoMineR*” package. We repeated the analysis across different branch order levels to evaluate whether the PCA results and the dimensions of the shoot economics space were sensitive to branch order.

We assessed whether branch orders associated with different levels of cortical chlorenchyma area (Order1, Order2, Order3, and Order4) differed significantly within the multidimensional principal component analysis (PCA) space—specifically, whether significant differences existed along the first two PCA axes. This was achieved using permutational multivariate analysis of variance (PERMANOVA), where the first two PCA axes were treated as response variables and branch order was treated as a fixed factor. Euclidean pairwise distances between branch orders in PCA space were used, and 999 permutations were performed using the “*pairwise.adonis*” function from the “*pairwiseAdonis*” package [39]. To test for the significance of differences among branch orders, we applied the false discovery rate (FDR) method to reduce the probability of Type I errors due to multiple comparisons [40]. All statistical analyses were performed using R v.4.1.3 (R Core Team, 2022).

## 3. Results

### 3.1. Trait Correlations in the Shoot

**Figure 3:**
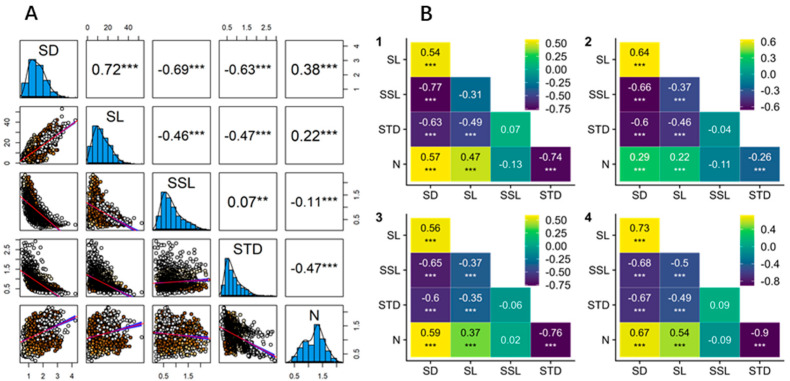
Pairwise correlations (Pearson’s r) among five functional traits of current-year shoots in *Larix principis-rupprechtii*. (**A**) All 1551 current-year shoots and (**B**) shoots by branch order: 1-Order 1 (*n* = 69), 2-Order 2 (*n* = 713), 3-Order 3 (*n* = 658), and 4-Order 4 (*n* = 111). Asterisks indicate significant correlations (** *p* < 0.01; *** *p* < 0.001). General linear regression was used to analyze bivariate relationships among functional traits across all current-year shoots (*n* = 1551). The scatterplots (lower triangle) show significant relationships with red regression lines (general linear regression). Correlation coefficients are shown in black in the upper triangle based on the linear regression analysis.

Here, we summarize the covariation patterns of functional traits in current-year shoots of *Larix principis-rupprechtii* across four different branch order levels. When considered in pairs, nearly all branch traits showed significant correlations both at the overall level and within individual branch orders (Figure 3). Specifically, SD and SL exhibited the strongest positive correlation (r = 0.72, Figure 3). Both SD and SL were negatively correlated with SSL (r = −0.69 and r = −0.43, Figure 3), while the relationship between SSL and STD was weak (r = 0.07, Figure 3). SD, SL, and branch N were all negatively correlated with STD (r = −0.63, r = −0.47, and r = −0.47; Figure 3).

### 3.2. The Proposal of the Conceptual Framework of the Shoot Economics Space

Although strong negative correlations have been observed between SSL and SD, STD and N, and SD and N, and a strong positive correlation has been observed between SD and N, the relationship between SSL and STD is weak and unclear. Correlation results across branch orders indicate that plant branches can construct branch systems with various combinations of SSL and STD, suggesting complex interactions among traits inconsistent with a one-dimensional branch economics spectrum.

**Figure 4:**
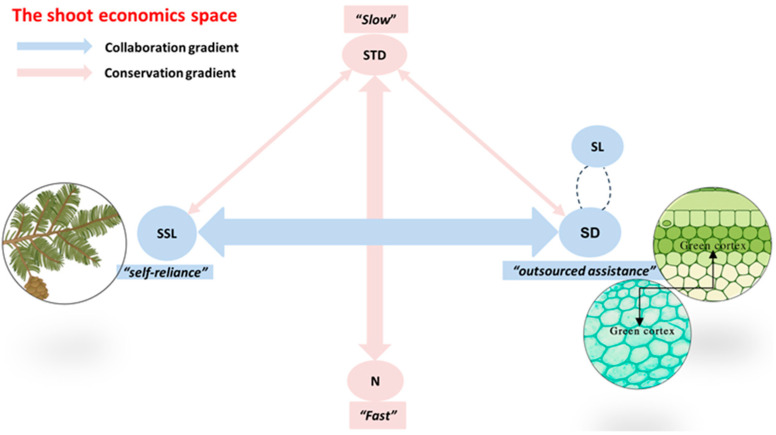
The conceptual framework of the shoot economics space.

By testing pairwise correlations among all functional traits of current-year shoots, we confirmed the bivariate relationships underlying the two main dimensions of the newly proposed concept of aboveground branch economic trait space (Figure 3). We found a strong negative correlation between SSL and SD (R = −0.69), representing a “cooperation” gradient ranging from self-reliance to outsourcing (Figure 4). We also observed the strongest negative correlation between STD and twig N (R = −0.84), consistent with findings from previous studies, which corresponds to a “conservation” gradient representing the traditional trade-off between fast and slow returns on investment (Figure 4).

Based on this concept, we hypothesize (i) a cooperation gradient, ranging from self-reliant leaf exploration characterized by high specific stem length (SSL) to outsourced acquisition via carbon investment into stem cortical chlorenchyma partners—and thus into chloroplast-derived photosynthates—which requires greater stem length (SL) and diameter (SD) and (ii) a conservation gradient, ranging from branches with high stem tissue density (STD), which exhibit slow returns on resource investment but have longer lifespans and greater structural protection, to fast-return branches with high nitrogen content (N) and metabolic rates but shorter lifespans. Arrows indicate negative correlations between individual traits (see Figure 3).

### 3.3. Multidimensional Coordination of Shoot Traits

**Figure 5:**
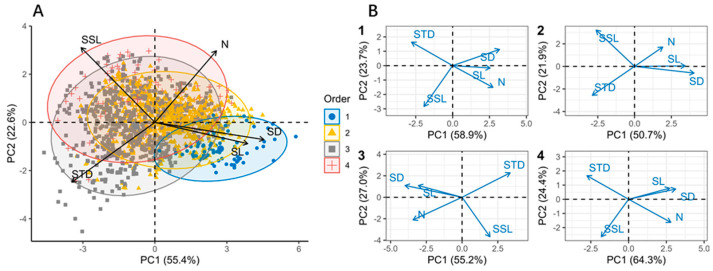
Principal component analyses (PCAs) based on functional traits showing (**A**) all 1551 current-year shoots and (**B**) shoots by branch order: “1” represents Order 1 (*n* = 69); “2” represents Order 2 (*n* = 713); “3” represents Order 3 (*n* = 658); and “4” represents Order 4 (*n* = 111). The cooperation gradient (55.4%) spans from high-SSL DIY branches to outsourced branches with a large stem diameter (SD). The conservation gradient (22.6%) spans from fast-return (N) to slow-return (STD) strategies. In (**A**), we highlight current-year shoots of four different branch order levels within each corner of the branch economics space: “1” stands for Order 1; “2” stands for Order 2; “3” stands for Order 3; and “4” stands for Order 4. PCA scores are shown in Table S1.

We went beyond pairwise trait comparisons to determine the degree of covariation among branch economic traits in a multidimensional trait space. This covariation can be quantified by the proportion of total trait variation explained by the first principal component in a principal component analysis (PCA). In a two-dimensional trait space, the principal axis represents the major axis of an ellipse formed by two correlated traits; a high proportion of variance explained by this axis effectively reflects the relationships among functional traits of current-year shoots (Figure 5). The principal axis explained 78.0% of the variation in the full dataset, and the cumulative variance explained by PCA axes 1 and 2 exceeded 70% across different branch orders. When branches were grouped by branch order, the directionality of trait loadings and similarly high percentages of variance explained by the principal axis indicated the broad generality of the branch economics space.

In a dataset of 1551 current-year shoots containing complete information on the five key branch traits (SD, SL, SSL, STD, and branch N), we were able to confirm these two distinct and largely independent gradients through principal component analysis (PCA). The first two axes defined a plane that cumulatively explained 78% of the total variation in branch traits. We therefore refer to these gradients as the primary dimensions of the “branch economics space” (Figure 5). The first PCA axis, accounting for 55.4% of the total trait variation, represents a gradient from SSL to SD, confirming our conceptualized cooperation gradient and showing that it is the dominant source of branch trait variation. The second PCA axis, explaining 22.6% of the total variation, represents the conservation gradient from branch N to STD (Table S1).

## 4. Discussion

### 4.1. Correlations in Shoot Traits Across Different Branching Orders

The currency of shoot economics is the carbon input required to construct fine shoots that explore and utilize leaf photosynthates to acquire resources [14,20,41]. Specific stem length (SSL)—the length of a shoot per unit mass—thus reflects the return on investment per unit of carbon and is a function of stem diameter (SD) and stem tissue density (STD), which together determine the mass per unit volume of a shoot [42].

$$\mathrm{SSL}=4/(\pi \times {SD}^{2}\times STD)$$

Although this equation is a simplification when sampling heterogeneous fine branching orders, it suggests that SSL increases as SD and/or STD decrease. In addition to efficiently exploring the resource sink of leaves, current-year shoots must maintain high metabolic rates to ensure rapid resource acquisition, resulting in high nitrogen content (N) in fine shoots [43].

The significant negative correlation between STD (stem tissue density) and SD (stem diameter) may stem from the fact that branches with high tissue density tend to have a lower vessel fraction, which can result in reduced transpiration, photosynthesis, and biomass growth rates [34,44,45]. Several studies have reported a significant negative correlation between branch tissue density and estimated mean diameter [27,30,46]. In comparisons across forest plots, slow diameter growth has been associated with high wood density, such as in the Amazon and Malaysia [27,46]. It is worth noting that in some cases, higher wood density may lead to greater annual growth rates. Given the numerous factors influencing growth and the different scales at which growth is measured, this result is not unexpected. Fibers and thick-walled vessels in dense wood can protect conduits from collapsing under the strong negative xylem pressure caused by water deficits [47]. If this adaptation holds true, it may allow heavy-wooded plant species growing in seasonally dry climates to continue growing even when light-wooded species are forced to shut down their water transport systems.

### 4.2. The Trade-Off Between Structural and Nutrient Investments

A growing body of literature suggests that the complexity of shoot traits may stem from the diversity of aboveground resource acquisition strategies [21,46,48,49,50]. Unlike photosynthesis, carried out solely by plant organs, many aboveground branches possess the ability to outsource resource acquisition. This gradient of plant branch cooperation strategies ranges from resource acquisition via self-reliant, cost-effective branches that explore efficiently with leaves, to outsourcing resource acquisition by investing carbon into chloroplast partners within the stem cortical chlorenchyma to obtain limited resources. However, these outsourcing strategies influence branch traits. This is especially true for current-year shoots of Order 1, where branches must increase their stem cortical chlorenchyma area—thus increasing their SD and SL values—to provide an internal habitat for chloroplast partners. This pattern applies to the symbiotic relationship between plant branches and chloroplasts in the stem cortical tissue. Building on this literature, we propose a general concept of branch economics based on the understanding that plants can optimize resource acquisition either by investing carbon in fine branches with efficient, self-reliant leaves or by forming efficient symbioses with stem cortical chlorenchyma (chloroplast) partners—an approach that typically requires more robust branches (Figure 4).

This conceptual cooperation gradient—from self-reliance to outsourcing—challenges the traditional one-dimensional spectrum of branch economics, which assumes that SD (stem diameter) increases with STD (stem tissue density) to achieve structural conservation. However, data show a negative correlation between SD and STD, likely due to the allometric growth relationship between branch anatomical components (i.e., cortex and stele). Analogous to the allometric rules observed in fine roots [51,52,53], as SD increases, cortex area expands faster than stele area, resulting in a positive correlation between SD and cortex fraction (CF), though this may vary across different branch order levels. Parenchyma tissue has lower carbon content and dry mass than stele tissue, which is responsible for nutrient and water transport via lignified cells [27]. Therefore, CF and STD are negatively correlated. Moreover, since SD and CF are closely related and increase in tandem with cortical chlorenchyma symbiosis, SD is negatively correlated with STD. These relationships refute the one-dimensional assumption of the branch economics spectrum—that slow-strategy plant branches are expected to be both thick and dense—and instead advocate for a multidimensional space of branch trait variation.

### 4.3. The Coordination of Economic Spectra Across Different Branching Orders

The current-year shoots associated with Order 3 and Order 4 are the two largest branching order categories in the dataset. They are distributed throughout the entire trait space (Figure 5A) but show significant differences compared to the shoots of Order 1 and Order 2 (Table S2). Shoots of Order 3 and Order 4 cluster at the DIY end of the cooperation gradient and the slow end of the conservation gradient. The variation in the cooperation gradient is smaller for current-year shoots of Order 1 than for those of Order 2, and they tend to be more outsourced and faster. High STD (indicating a slow strategy) may partly result from the primary function of shoots associated with Order 3 and Order 4 being mechanical support [54] and may also reflect the generally slow nutrient cycling in ecosystems dominated by higher-order current-year shoots [55]. Order 3 and Order 4 tend to have high-SSL DIY current-year shoots, which not only arises from their lower dependence on stem cortical chlorenchyma area [56] but also relates to the hierarchical investment between vegetative growth and reproductive resources at the branch level [36]. Specifically, lower-order terminal branches grow more but rarely flower, while in higher-order branches, shoot growth is heavily constrained regardless of reproductive intensity, leading to resource stress. In other words, current-year shoot growth may be preferentially allocated to lower-order shoots, possibly due to differences in the opportunity cost of reproduction across branching orders. Nevertheless, principal component analysis (PCA) of the functional traits of shoots associated with Order 1, Order 2, Order 3, and Order 4 reveals the same axes of variation as those found in the overall plant dataset (Figure 5B, 1, 2, 3, and 4, as well as Table S1), highlighting consistent trade-offs within each branch order level.

Lower-order shoots associated with stem cortical chlorenchyma (chloroplasts) differ from higher-order shoots (Figure 5A), as they lie on one side of the conservation gradient due to their branching systems being nitrogen-rich (Figure 5A). Nevertheless, we can confirm that the cooperation gradient is the primary principal component axis for this species group (Figure 5A and Table S1). The importance of the cooperation gradient in current-year shoots may stem from the high nitrogen and phosphorus demands of shoots associated with chloroplast-rich stem cortical tissue, which leads shoots to either rely heavily on the chlorenchyma or adopt alternative DIY strategies, as seen in higher-order shoots. When examining different branch order levels, we found that higher-order shoots exhibit a wider range of variation in regional trait space than lower-order shoots (Figure 5A and Table S1). However, both gradients of the shoot economic space are present in both higher- and lower-order shoots (Figure 5 and Table S1), indicating widespread variation and very similar trade-offs regardless of branch order. Finally, the two dimensions of the shoot economic space are consistently present in current-year shoots (Figure 5 and Table S1), even if their positions in regional trait space differ (Figure 5 and Table S1).

## 5. Conclusions

We provided a conceptual framework to explain the mechanistic basis behind the covariation of branch system functional traits in *Larix principis-rupprechtii* and demonstrated its universality across different branch order levels. The shoot economic space, integrating the latest data, shows that variation in shoot traits cannot be fully explained by a single-dimensional spectrum. Plants acquire aboveground resources through collaboration with their stem green cortex (chloroplast) partners, which is not merely an “extra” dimension in the shoot economic space but rather the primary axis of shoot trait variation—fundamentally different from the leaf economics spectrum (LES). The gradient from self-reliance to outsourced cooperation represents the investment in resource acquisition either by the branch system itself or by its stem green cortex (chloroplast) partners. This gradient is independent of the protection gradient, which represents the well-known concept of the speed of return on investment. Therefore, these two gradients depict different aspects of shoot economics, encompassing the entire shoot economic space for plant aboveground resource acquisition, rather than a single one-dimensional spectrum. Our key findings from this single species, *Larix principis-rupprechtii*, should encourage further research to elucidate and test whether the shoot economics spectrum is universal across other plant species.

## Data Availability

The data that support the findings of this study are available from the corresponding author or the first author upon reasonable request.

## Supplementary Materials

The following supporting information can be downloaded at https://www.mdpi.com/article/10.3390/life15081310/s1. Table S1: Summary of principal component analysis of functional traits of current-year shoots across branch orders; Table S2: Permutational multivariate analysis on four branching orders displaying variation in five functional traits.
