# Supplementary material for: The Cortical Chlorenchyma Collaboration Gradient Dominates the Shoot Economics Space in Larix principis-rupprechtii
Source: Life (Basel). 2025 Aug 19;15(8):1310. doi: 10.3390/life15081310 (PMC12387127; doi:10.3390/life15081310)
Supplement: Supplementary file 1 [file life-15-01310-s001.zip › life-3808905-supplementary.pdf]

Table S1. Summary of principal component analysis of functional traits of current-year shoots across branch orders

	All	Order1	Order2	Order3	Order4
**Variation explained (%)**	78.0	82.5	72.6	82.3	88.7
**Shoot trait**	**Loadings**				
SD (mm)	0.57	0.53	0.59	0.55	0.52
SL (cm)	0.48	0.43	0.50	0.42	0.45
SSL (m/g)	-0.39	-0.33	-0.38	-0.27	-0.30
STD (g/cm3)	-0.43	-0.47	-0.42	-0.47	-0.46
N (mg/g)	0.32	0.46	0.28	0.47	0.47

Table S2. Permutational multivariate analysis on 4 branching orders displaying variation in five functional traits, including shoot diameter (SD)、length (SL)、specific stem length (SSL)、specific stem density (STD), and Nitrogen (N). 1-Oder1 (n=69), 2-Order2 (n=713), 3-Order3 (n=658), 4-Order4 (n=111). *$p < .05$; **$p < .01$; ***$p < .001$.

Pairs	Sums of squares	F	R^2	P
**1vs2**	18810.98	361.57	0.316	0.006
**1vs3**	27510.42	717.19	0.497	0.006
**1vs4**	22613.42	732.69	0.805	0.006
**2vs3**	4761.38	102.91	0.070	0.006
**2vs4**	3200.76	64.13	0.072	0.006
**3vs4**	433.39	11.77	0.015	0.006
